# Predictive Models of Primary Tropical Forest Structure from Geomorphometric Variables Based on SRTM in the Tapajós Region, Brazilian Amazon

**DOI:** 10.1371/journal.pone.0152009

**Published:** 2016-04-18

**Authors:** Polyanna da Conceição Bispo, João Roberto dos Santos, Márcio de Morisson Valeriano, Paulo Maurício Lima de Alencastro Graça, Heiko Balzter, Helena França, Pitágoras da Conceição Bispo

**Affiliations:** 1 Ciência e Tecnologia Ambiental, Universidade Federal do ABC (UFABC), Santo André, São Paulo, Brazil; 2 Centre for Landscape and Climate Research, Department of Geography, University of Leicester, Leicester, United Kingdom; 3 Divisão de Sensoriamento Remoto, Instituto Nacional de Pesquisas Espaciais (INPE), São José dos Campos, São Paulo, Brazil; 4 Coordenação de Dinâmica Ambiental, Instituto Nacional de Pesquisas da Amazônia (INPA), Manaus, Amazonas, Brazil; 5 National Centre for Earth Observation, University of Leicester, Leicester, United Kingdom; 6 Departamento de Ciências Biológicas, Faculdade de Ciências e Letras de Assis, Universidade Estadual Paulista (UNESP), Assis, São Paulo, Brazil; Trier University, GERMANY

## Abstract

Surveying primary tropical forest over large regions is challenging. Indirect methods of relating terrain information or other external spatial datasets to forest biophysical parameters can provide forest structural maps at large scales but the inherent uncertainties need to be evaluated fully. The goal of the present study was to evaluate relief characteristics, measured through geomorphometric variables, as predictors of forest structural characteristics such as average tree basal area (BA) and height (H) and average percentage canopy openness (CO). Our hypothesis is that geomorphometric variables are good predictors of the structure of primary tropical forest, even in areas, with low altitude variation. The study was performed at the Tapajós National Forest, located in the Western State of Pará, Brazil. Forty-three plots were sampled. Predictive models for BA, H and CO were parameterized based on geomorphometric variables using multiple linear regression. Validation of the models with nine independent sample plots revealed a Root Mean Square Error (RMSE) of 3.73 m^2^/ha (20%) for BA, 1.70 m (12%) for H, and 1.78% (21%) for CO. The coefficient of determination between observed and predicted values were r^2^ = 0.32 for CO, r^2^ = 0.26 for H and r^2^ = 0.52 for BA. The models obtained were able to adequately estimate BA and CO. In summary, it can be concluded that relief variables are good predictors of vegetation structure and enable the creation of forest structure maps in primary tropical rainforest with an acceptable uncertainty.

## Introduction

Deforestation and forest fragmentation by human activities are some of the main environmental problems of tropical forests. The destruction and degradation of the forest cover decreases its ecological integrity, decreasing biodiversity and negatively impacting the functioning of these systems [1,2]. Therefore, the search for indicators and models capable of detecting spatial and temporal variations of forest structure in natural environments is highly relevant, enabling natural variability to be distinguished from changes of anthropic origin. Models based on reference conditions (natural areas or with minimum impact) allow estimation of the expected vegetation over a gradient of different vegetation types in undisturbed forests. The difference between the expected characteristics of the natural vegetation for a given region and those observed may be considered an indicator of the intensity of anthropic impact on the forest cover.

Forest structure can affect several ecosystem processes [3,4,5] and biodiversity [6,7,8], as it affects nutrient cycling [9] and the availability of niches for several species [10,11]. Therefore, forest structure characteristics are likely good indicators of forest functioning and biodiversity [8,9,12,13]. In addition, vegetation structure characteristics may give information regarding biomass, which is essential for carbon stock quantification, and its monitoring allows the evaluation of the effectiveness of measures for reducing emissions from deforestation and forest degradation (REDD+).

Average tree basal area (BA) and height (H) of trees and average percentage of canopy openness (CO) are often used to describe forest structure. Together, these parameters can offer a general view of the vegetation structure, which can be determined from different environmental factors, namely relief, soil, temperature, water availability, geological structure or fire incidence [14,15,16,17,18,19,20,21]. Among these factors, the relief underneath a forest canopy is usually not changed significantly by anthropic activities and is therefore thought to be very effective for the construction of predictive models for the estimation of vegetation characteristics in natural areas. Moreover, relief may affect other equally important factors (e.g. soil characteristics); hence, it may have direct or indirect effects on vegetation.

In the present study relief was represented by local geomorphometric characteristics, such as elevation, slope gradient and slope aspect, which strongly affect vegetation structure [16,22,23]. Elevation (height of land relative to sea level) and its variation are usually related to local microclimate. Slope gradient and slope aspect determine the intensity and direction of flows of matter and insolation, respectively, and affect the local water and energy regimes. In addition to these variables, the basic set of characteristics for local land characterization should also include the plan and profile curvatures [22,24,25,26]. The plan curvature corresponds to the variation among the convex, straight and concave landforms of the terrain and the profile curvature corresponds to the variation among the divergent, neutral and convergent landforms of the terrain [27]. These two variables combined characterize the land surface, which is directly associated with hydrological and transport properties and may influence vegetation indirectly [26,28].

Several studies have analysed the influence of environmental variability on spatial differentiation of vegetation. However, few have rigorously tested the effects of local geomorphometric variation on the structural characteristics of vegetation [22,29,30], such as BA, H and CO, especially when the relief descriptor variables are derived from remote sensing data. The topography can affect the vegetation structure both directly and indirectly. For example, Webb et al. [31] analysed the direct effects of topography on forest structure in an area of American Samoa and observed higher tree density in ridge forests, with trees of wide diameter and low height at higher altitudes (usually in ridges). In contrast, relief also influences other variables that are important for vegetation, such as humidity, nutrient availability, solar radiation, temperature and soil characteristics, which may have indirect effects on vegetation [21,22,32,33,34,35].

As geomorphometric variables are available from satellite missions such as the Shuttle Radar Topography Mission (SRTM), they offer a methodological approach for indirect estimation and mapping of forest structural variables at larger scales, if robust relationships between the two are confirmed. The magnitude of the uncertainties in forest structure maps from geomorphometric proxies needs to be carefully quantified. Thus, because relief characteristics are related to vegetation structure [29,36], the goal of the present study was to evaluate topographic characteristics through measurement of local geomorphometric variables as predictors of structural characteristics of vegetation (BA, H and CO). Our hypothesis is that geomorphometric variables are good predictors of the vegetation structure of a primary tropical forest, even in an area with low altitude variation. If this relationship is confirmed, models will be built and used to generate maps.

## Material and Methods

### Study area

**Fig 1:**
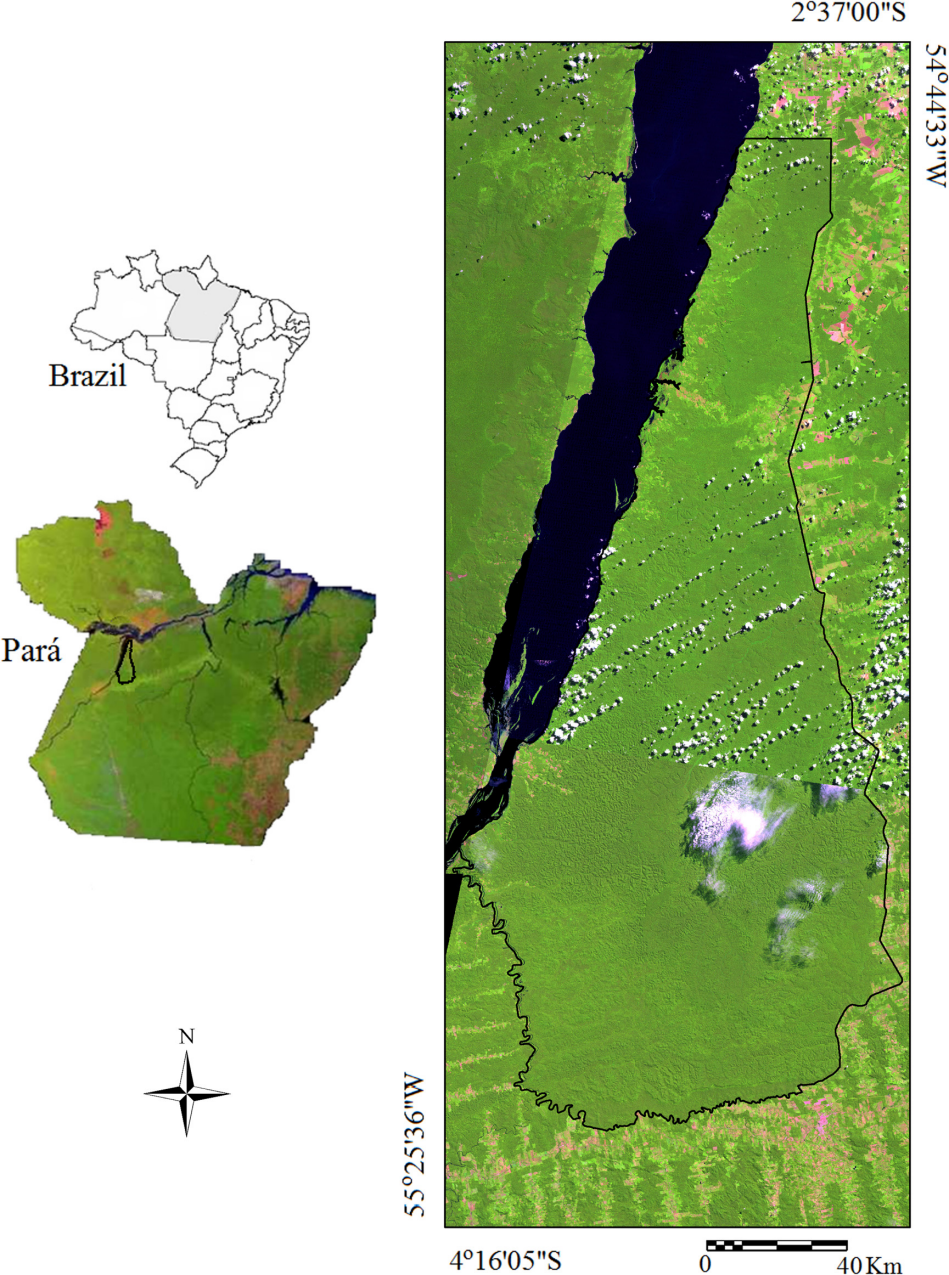
Study area in the Tapajós National Forest.

The study area belongs to the Tapajós National Forest (TNF), between coordinates 2°35’ to 4°20’S and 54°40’ to 55°40’ W, located in the Lower Amazon River mesoregion, Western of Pará State, Brazil (Fig 1). The TNF is an area of environmental protection with approximately 545.000 ha representative of Amazon Forest.

The region's climate is Ami type, according to the Köppen climate classification, with approximately 25°C annual average temperature, 1800 mm annual average rainfall, and higher rainfall concentration between January and May [37]. The predominant soil types at the area are Yellow Oxisol and Red-Yellow Ultisol. The vegetation is mostly classified as Dense Ombrophilous Forest and Open Ombrophilous Forest [38].

### Field data

A survey of the forest structure was performed in 43 plots with 25 x 100 m^2^, in a total area of 10.75 ha. In the present study, the surveyed plots presented different topographic positions and phyto-ecological classes, defined according to floristic, lithological and geomorphological characteristics of the TNF [39].

The vegetation typology in this area consist mostly of dense tropical rain forest and a small portion of open tropical rain forest in the southwest area of TNF. Within the 10.75 ha total sampled area, the elevation varies between 81 and 218 m, whereas slope varies from 2 to 27%. In total, 4163 arboreal individuals (49 plant families and 232 species) were found during the field work. Bispo et al. [28] in their study about the effects of the geomorphometric characteristics of the local terrain on floristic composition in the central Brazilian Amazon showed more details about the floristic of the study area.

The geographic position of diameter at breast height (DBH) and total height (H) were measured for all tree individuals with DBH ≥ 10 cm in each plot. Averages diameter at breast height, averages at height and canopy openness (CO) were therefore recorded for all plots. Tree basal area (BA) was calculated using DBH according to the following equation:

$$\text{BA}=\sum (\pi \;*{\left(\text{DBH}/2\right)}^{2})$$

Tree height (H) was visually assessed by a trained specialist. To improve visual estimates, these data were adjusted using the equation developed by Gonçalves et al. [40], which was based on the measurement of 277 trees at the study area and relates visual assessment of tree height with measurements performed using an electronic clinometer:

$$\text{Hce}={\text{e}}^{0.1845}{\text{Hev}}^{0.9480}$$

Where Hce represent the tree height measured using an electronic clinometer and Hev represent tree height visually assessed.

**Fig 2:**
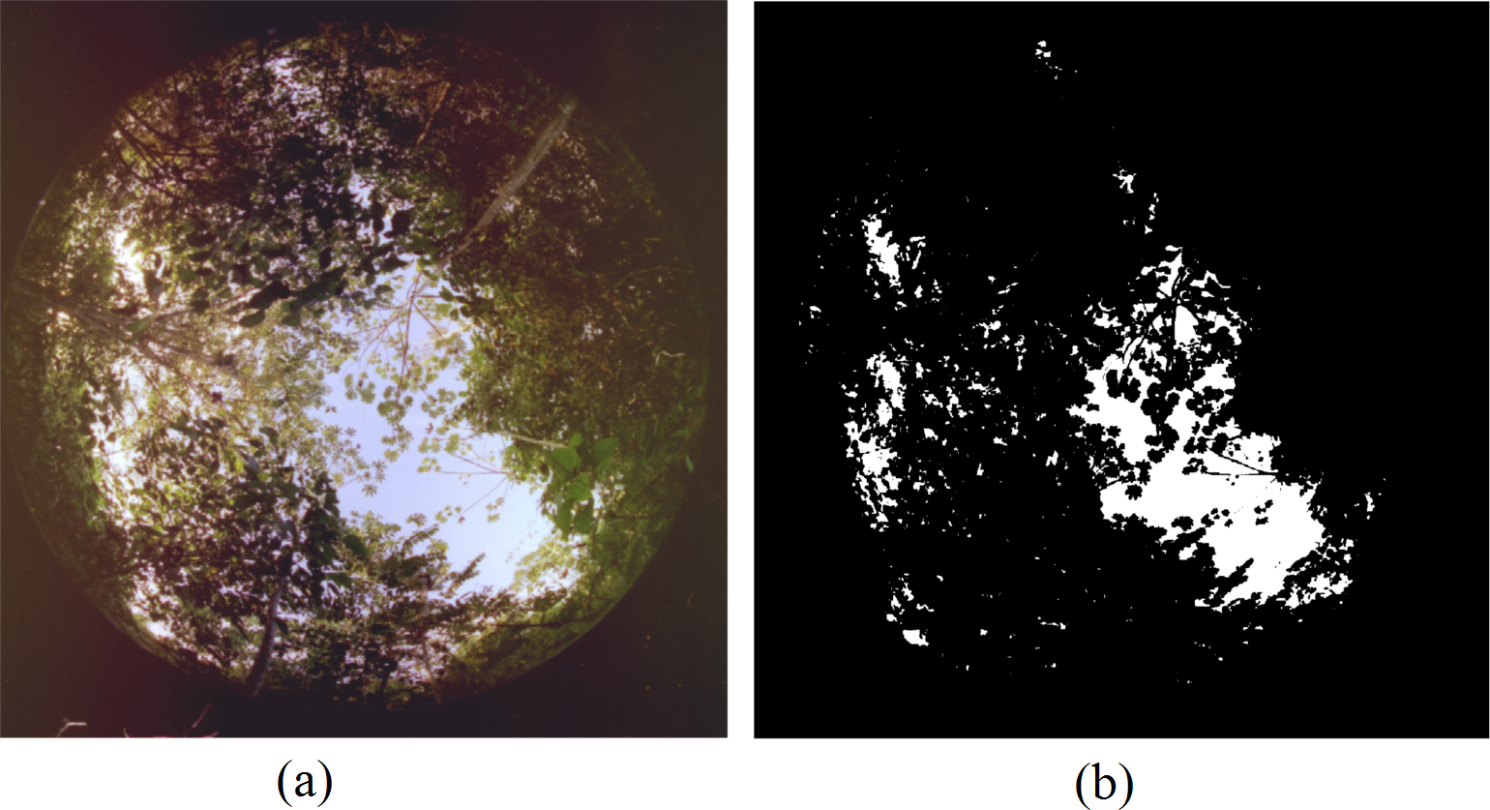
Illustration of (a) a hemispherical photograph of forest cover and (b) a binary image resulting from GLA analysis.

DBH was measured using a measuring tape. CO was estimated from hemispherical images obtained using a digital camera with 10.2 Megapixel resolution (Nikon D60) coupled to a wide-angle lens (fisheye) (Soligor 0.25x52 mm). The images were captured 1.8 m from the ground, with a 90 degree angle relative to the ground, under the canopy and avoiding direct sunlight, i.e., under conditions of uniform sky before sunrise (from 6:30 to 8:30 am) or after sunset (3.30 to 5:00 pm). These images were taken along central axis transects (25 x 100 m), every 20 m (following the same method used by Galvão et al. [41]), totaling in 5 images per transect. Images were analysed using Gap Light Analyser software (GLA) [42], and the fraction of canopy openness (Gap fraction) was calculated. Gap fraction is defined as the percentage of canopy gaps, i.e., the probability of sunlight passing through the forest canopy without meeting leaves or another plant part [43]. The GLA package uses an image classifier based on pixel thresholds to calculate the fraction of canopy openness. Therefore, a threshold needs to be defined for each image, which can vary with sun exposure. The images of the canopy and sky were adequately converted and separated into black and white pixels. The resulting images in binary format were used for determining the fraction of canopy openness (Fig 2).

To facilitate data integration, analysis and to enable the performance of spatial queries, the dendrometric parameter data were structured using the Geographic Information System (GIS).

### Geomorphometric data derived from SRTM/TOPODATA

The geomorphometric data used (i.e., elevation, slope gradient, slope aspect, plan and profile curvature) were obtained from the Brazilian Geomorphometric Data Bank (TOPODATA) [44]. For the analysis, we used the TOPODATA that are based on the SRTM (Shuttle Radar Topography Mission—version 1, NASA, 2006) () refined for the Brazilian territory from the original resolution of 3 arc seconds to 1 arc second using a geostatistical approach [45]. Different neighborhood operations were applied to calculate geomorphometric variables [46].

There was a preference to use TOPODATA instead of the original SRTM. as it includes the derivations calculated with algorithms specially developed for the SRTM characteristics, which were widely tested on varied reliefs in order to run uniformly on the full extension of the Brazilian territory [46] In addition, TOPODATA is also free and their layers are easily accessible for their use in GIS (http://www.dsr.inpe.br/topodata/acesso.php). The geomorphometric variables obtained from TOPODATA are presented in Table 1. Subsequently, values for the different variables analysed were extracted for each of the surveyed plots and used for modeling the forest structure.

**Table 1:** Definitions of the topographic variables used in this study.

Geomorphometric variables	Description
Elevation (*h)*	Terrain altitude; it is related to the altitude distribution of soil and climate, determining different landscape vegetation patterns.
Slope gradient (*G)*	Inclination angle of the local surface; has a direct effect on the balance between soil water infiltration and surface runoff and controls the intensity of flows of matter and insolation. This set of factors results in environments with different physical and biological characteristics, allowing the establishment of different types of vegetation.
Slope aspect (*A)*	Terrain alignment relative to the sun; it is the horizontal angle relative to the expected direction of surface runoff, usually expressed in azimuth. Among several aspects (i.e., relationships with the distribution of different substrates, ecological niches, etc.), this variable is related with the degree of shade or light in the terrain, selecting more appropriate environments for the development of certain types of vegetation. The slope aspect corresponds to the angle from 0° to 360°. Since aspect is a circular variable, it was converted in two linear components given by sine/cosine transformation generating two new variables *Sine A* (Sine of slope aspect) and *Cosine A* (Cosine of the slope aspect) which were used for regression analyses instead.
Profile curvature (*kv*)	Concave/convex character of the terrain. This characterizes the land surface, which is directly associated with hydrological and transport properties and may influence the distribution and development of vegetation indirectly.
Plan curvature (*kh)*	Divergent/convergent character of flows of matter on the ground when analysed on a horizontal projection. As the profile curvature, the plan curvature characterizes the land surface, which is directly associated with hydrological and transport properties and may influence vegetation indirectly.

### Processing of information layers, data extraction and pairing

Geomorphometric data layers included elevation, slope, aspect and profile and plan curvatures, as described. Since aspect is a circular variable, its linear components given by sine/cosine transformation were used for regression analyses instead.

Local geomorphometric data were obtained from the TOPODATA in GeoTIFF format, from the elevation image (Digital Elevation Model—DEM refined) and its derivations. For each variable, the grids (1° x 1.5° each) covering the study area were concatenated into an image mosaic.

The two datasets (vegetation and geomorphometric data) were overlaid after matching their respective georeference systems by reprojecting the raster images and plot polygons to a common projection. Geomorphometric data were averaged within plot extents and extracted in correspondence to vegetation data, resulting in paired datasets for further analysis in statistical software environments.

### Statistical modeling of structural data

Predictive models were generated for each variable (BA, H and CO) based on local geomorphometric variables using multiple regression analyses [47]. Independent variables were selected using the forward stepwise selection procedure. A preliminary analysis showed the presence of four outliers in the observation group. These outliers were excluded from the analyses, and a new evaluation using Cook's distance was performed, showing the absence of additional outliers. Fitting of regression models and subsequent steps were performed following the recommendation that the total number of observations should be 10 times the number of variables [47]. In the present study, this result corresponded to a maximum of 3 explanatory variables. Analyses were performed to ensure that there were no violations of the assumptions of normality and homoscedasticity, and of no spatial autocorrelation. The presence of multicollinearity was tested using the variance inflation factor (VIF) and the autocorrelation was tested using Moran's Index [48].

The models were built using thirty observations and were validated using nine independent observations. Model validation was performed by comparing observed and model-predicted values using the RMSE (Root Mean Square Error) [47] and the coefficient of determination (r^2^).

## Results

The models obtained were statistically significant. The data analysed showed VIF < 10, indicating the absence of serious problems with multicollinearity [47] (Table 2). The residuals showed homogeneity of variance (Levene test, *p*>0.05) and did not present spatial autocorrelation for any distance class according to Moran's Index (*p*>0.05). The plot of residuals against predicted values showed that the linearity and homoscedasticity assumptions were met.

**Table 2:** Multiple regression analysis of the relationships between BA (basal area), CO (canopy openness) and H (height) and local geomorphometric variables selected by stepwise method (*h*: elevation, *Cosine A*: Cosine of slope aspect, *G*: slope gradient and *kv*: profile curvature). As the coefficients *β* had *p* < 0.1 for all variables selected, thus all of them were included in equations models.

Variable	*β*	*SE*	*t*	*p*	*VIF*
**Model BA**					
Constant	-1.53	3.44	-0.44	0.65	
*h*	0.13	0.01	7.71	0.00	1.12
*G*	0.28	0.12	2.21	0.03	1.12
r² = 68.82%; BA = -1.53 +0.13*h* +0.28*G*					
**Model H**					
Constant	15.77	0.47	33.08	0.00	
*G*	0.078	0.03	2.17	0.03	1.00
*kv*	15.60	10.50	1.50	0.09	1.00
r^2^ = 20.33%; H = 15.77 + 0.078 *G* + 15.60 *Kv;*					
**Model CO**					
Constant	18.32	1.91	9.6	0.00	
*h*	-0.07	0.012	-5.72	0.00	1.02
*Cosine A*	1.69	0.92	1.83	0.079	1.01
*kv*	63.40	26.50	2.40	0.024	1.03
r^2^ = 60.85%; CO = 18.31 - 0.07 *h* + 1.69* cos A* + 63.40 *kv;*					

The model for estimating BA presented explained 68% of the variance in basal area (r^2^ = 0.68, Adjusted r^2^ = 0.66, F_2.27_ = 29.809, *p*<0.001 and Std. Error of Estimate = 4.1207) and included the explanatory variables elevation and slope gradient (Table 1). The model of H, despite being significant, explained only 20% of the variance (r^2^ = 0.20, Adjusted r^2^ = 0.14, F_2.27_ = 3.44, *p*<0.047 and Std. Error of estimate = 1.236) and included the variables profile curvature and slope gradient (Table 1).The model of CO presented explained 60% of the variance (r^2^ = 0.60, Adjusted r^2^ = 0.56; F_3.26_ = 13.46, *p*<0.001 and Std. Error of estimate = 3.088) and included the variables elevation, cosine of the orientation slope, and profile curvature (Table 1).

**Fig 3:**
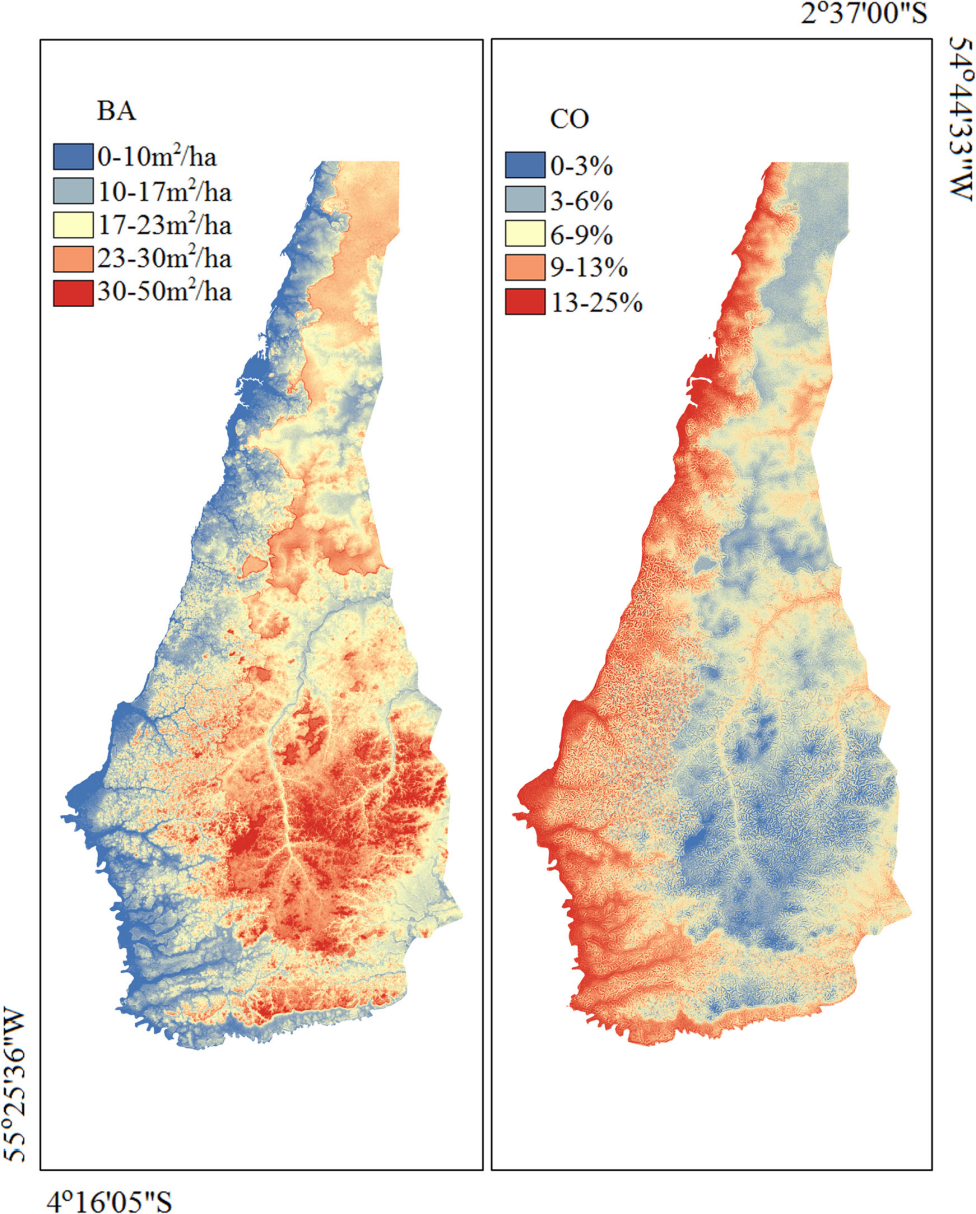
Maps of estimated BA and CO for Tapajós National Forest.

Model validation with 9 independent sample plots showed an RMSE of 3.73 m^2^/ha (20%) for BA, 1.70 m (12%) for H, and 1.78% (21%) for CO relative to the average values. The coefficients of determination from the analyses between observed and predicted values were r^2^ = 0.32 for CO, r^2^ = 0.26 for H and r^2^ = 0.52 for BA. Because the coefficient of determination of the modelled H with 30 samples was low and that one generated with the independent observations to this structural variable was not significant, we considered the obtained model inadequate for estimation of this variable. Following validation, the models for BA and CO were applied, and maps for these two variables were generated for TNF (Fig 3).

The generated models and maps indicated that individuals with a higher basal area were located in areas with higher elevation and slope gradient. More closed canopies were associated with areas with higher elevation and slope aspect mostly facing South (lower slope aspect cosine) (Table 1, Fig 3).

## Discussion

Our data have revealed that topographic relief is a good predictor of certain forest structural characteristics. Of the three geomorphometric characteristics measured, average basal area (BA) and average canopy openness (CO) were adequately predicted by relief, although comparing the results we can notice that the model generate by BA is adjusted better than CO. For average height (H), although the error was low (RMSE = 12%), the r^2^ for the model was only approximately 26%, meaning that only a small proportion of the variability in H was explained by the model. In this case, considering the 9 independent validation sample plots, the coefficient of determination between observed and predicted H values was not significant. Therefore it can be considered that the multiple regression models were not satisfactory for this variable. In summary, we conclude that the relief variables used were only sufficient to adequately estimate BA and CO. In the case of H, including additional variables in the model, such as the type of soil, geological structure and water availability, may improve its predictive capacity.

The basal area estimates included the variables altitude and slope gradient, whether the canopy openness estimates included altitude, profile curvature and cosine of slope aspect. Of these variables, altitude may be considered the one variable with higher predictive capacity of vegetation structure, since its variation was highly consistent with the spatial variation of the basal area and canopy openness estimates. The estimates generated by the models revealed that sites presenting higher CO also presented tree individuals with lower BA and that higher CO and lower BA occurred in areas with lower altitude. Therefore, we suggest that altitude is the main predictor variable of spatial macro-variation of forest structure in the study area. The remaining variables included in the models (slope gradient, cosine of slope aspect and and profile curvature) seem to be related with minor components of this variation.

Relief characteristics, such as those evaluated through geomorphometric variables in the present study, have previously been considered as determinants of vegetation structure in forest ecosystems [28,29,36,49]. These characteristics may affect vegetation structure directly or indirectly, for example by affecting the control of temperature [50] and incident sunlight, general soil characteristics [51,52], flow of water and organic matter, and nutrient cycling [53]. Because the altitude variation in the TNF is low (approximately 300 m), the indirect effects of relief on soil characteristics must have been more important for determination of vegetation structure than the direct effects. The TNF topography seems to influence soil texture, with higher areas presenting predominance of clay and, consequently, higher humidity and carbon and nitrogen concentrations [54]. Moreover, the lower altitude terrains close to the Tapajós River are sandier [54]. Therefore the interaction between relief and soil is also likely to play an important part in determining the structural variation of vegetation within the TNF through edaphic processes that determine the soil catena.

Regions closer to the Tapajós River present Dystrophic Yellow Oxisol with medium texture, from clayey to sandy (dystrophic Entisols), dense forest/savanna transition, and flat to mildly hilly relief. Due to the dense forest/savanna transition, these areas present a slightly lower tree density. The models generated in the present study showed higher CO and individuals with lower BA in these areas. These forest characteristics can also be found in areas more to the southwest and extreme south of the TNF, with the occurrence of more open forests.

In contrast, TNF areas with higher altitude generally presented higher BA and lower CO. As previously stated, these areas present more clayey soils and, consequently, higher carbon and nitrogen concentrations, which enables higher vegetation development. These results are corroborated by Castilho et al. [49], who studied the Adolpho Ducke Forest Reserve, located in the Central Amazon, and observed positive correlations between forest biomass and the soil texture gradient (clay concentration) and altitude. These authors observed that the biomass of larger trees tends to be higher in clay-rich soils (more frequent in flat areas with higher altitudes) than in more sandy soils (low altitudes and bottom lands). In this area of the Central Amazon, the soil clay content is highly related to altitude (r = 0.94). As the clay content decreases, the sand content increases along the terrain succession from plateaus to valleys [55].

The relationships discussed above show that most effects of relief on vegetation can be explained by their relationships with the soil type, especially when different altitudes are compared. Different soil types present important nutritional differences, which could partly explain the vegetation structure variation. For example, in general, greater forest structure or biomass is expected in more fertile soils, independently of the species composition, simply because there are more resources available to support plant growth [56]. These factors could explain the observed higher basal area and lower canopy openness in sites with higher altitude, where soil tends to be more clayey in the TNF. Furthermore, different geological substrates are disposed in strata according to altitude, established during the sedimentation process, which could partly explain the relationship between soil and elevation.

The intensity of the impact of human activities on vegetation structure can be evaluated by comparing pre-existing and current vegetation [57,58]. Considering the complexity of the Amazon tropical forest, developing models that can predict expected vegetation based on reference conditions (natural environments or with minimum impact) is a challenge, especially with the goal of large-scale mapping. Therefore, we suggest the construction of more models of this type, following an approach of compartmentalization according to environmental mesoregions. These models are more useful if they are based on predictive variables that are little impacted by anthropogenic activities, such as relief, allowing the prediction of forest structure prior to disturbance. The present study shows that the use of relief variables results in good predictive models of the tropical forest structure, especially for canopy openness and tree diameter characteristics in the case of the FNT, allowing the elaboration of maps with acceptable uncertainty intervals ofabout 20%. In the Tapajós National Forest the uncertainty levels found for modeling biomass, forest height and volume has ranged from 10–25% [32,43,59]. In our study, for the canopy openness the level of uncertainty found was 21% and to the basal area was 20%.

Future steps to refine this variable integration for other Amazon mesoregions are needed to increase knowledge and to supply tools to help with control and supervision policies for the environmental monitoring organs of this region. The availability of satellite-derived global digital terrain models such as SRTM and the TanDEM-X DEM, which is currently in production, are a valuable resource for the indirect mapping of forest structural parameters in primary tropical forest. These maps will be useful for improving regional or global monitoring of forest structure and biodiversity in Amazon Biome.

## Authorization for the field work

The study was carried out in the Tapajós National Forest (TNF) and dendrometric measurements (diameter at breast height and height) as well as botanical identification of the trees were done, just inside of this area. The authorization to carry out the field work at TNF was provided by the Instituto Chico Mendes de Conservação da Biodiversidade-ICMBio/MMA (SISBIO n. 20591–1). This study did not involve endangered or protected species and no biological samples were taken.

## Supporting Information

S1 File Graphics of Morans’ Index. (DOCX)

S1 Table Data to generate the regression models. (XLSX)

S2 Table Data to validate the models. (XLSX)

## References

[1] Fahrig L. Effects of habitat fragmentation on biodiversity. Annu. Rev. Ecol. Evol. System. 2003; 34:487–515.

[2] Rawi CSM, Al-Shami SA, Madrus MR, Ahmad AH. Local effects of forest fragmentation on diversity of aquatic insects in tropical forest streams: implications for biological conservation. Aquatic Ecology. 2013; 47:75–85.

[3] Running SW, Coughlan JC. A general model of forest ecosystem processes for regional applications I. Hydrologic balance, canopy gas exchange and primary production processes. Ecological Modelling. 1988; 42(2):125–154.

[4] Lauenroth WK, Urban DL, Coffin DP, Parton WJ, Shugart HH, Kirchner TB et al Modeling vegetation structure-ecosystem process interactions across sites and ecosystems. Ecological Modelling. 1993; 67:49–80.

[5] Cramer W, Bondeau A, Woodward FI, Prentice IC, Betts RA, Brovkin V et al Global response of terrestrial ecosystem structure and function to CO2 and climate change: results from six dynamic global vegetation models. Global Change Biology. 2001; 7:357–373.

[6] Boncina A. Comparison of structure and biodiversity in the Rajhenav virgin forest remnant and managed forest in the Dinaric region of Slovenia. Global Ecology and Biogeography. 2000; 9(3):201–211.

[7] Pardini R, Souza SM, Braga-Neto R, Metzger JP. The role of forest structure, fragment size and corridors in maintaining small mammal abundance and diversity in an Atlantic forest landscape. Biological Conservation. 2005; 124(2):253–266.

[8] Gao T, Hedblom M, Emilsson T, Nielsen AB. The role of forest stand structure as biodiversity indicator. Forest Ecology and Management. 2014; 330:82–93.

[9] Pereira HM, Ferrier S, Walters M, Geller GN, Jongman RHG, Scholes RJ et al Essential Biodiversity Variables. Science. 2013; 339:277–278. doi: 10.1126/science.1229931 23329036 10.1126/science.1229931

[10] Giller PS. Community Structure and the Niche. London: Chapman and Hall; 1984.

[11] Diaz L. Influences of forest type and forest structure on bird communities in oak and pine woodlands in Spain. Forest Ecology and Management. 2006; 223(1–2):54–65.

[12] Noss RF. Indicators for Monitoring Biodiversity: A Hierarchical Approach. Conservation Biology. 1990; 4(4):355–364.

[13] Orians G, Dirzo R, Cushman JH. Biodiversity and Ecosystem Processes in Tropical Forests. Springer-Verlag, Berlin and Heidelberg, GmbH and Co. K, 220 pages; 2011.

[14] Franklin J. Predictive vegetation mapping: Geographic modelling of biospatial patterns in relation to environmental gradients. Progress in Physical Geography. 1995; 19(4):474–499.

[15] Chen ZS, Hsieh CF, Jiang FY, Hsieh TH, Sun IF. Relations of soil properties to topography and vegetation in a subtropical rain forest in southernTaiwan. Plant Ecololy. 1997; 132:229–241.

[16] Wilson JP, Galant JC. Terrain analysis: principles and applications New York: John Wiley and Sons; 2000.

[17] Catterall CP, Piper SD, Bunn S E, Arthur J. M. Flora and fauna assemblages vary with local topography in a subtropical eucalypt forest. Austral Ecology. 2001; 26: 56–69.

[18] Fisk MC, Schimidt SK, Seastedt TR. Topographic patterns of above and belowground production and nitrogen cycling in alpine tundra. Ecology. 1998; 79:2253–2266.

[19] Tateno R, Takeda H. Forest structure and tree species distribution in relation to topography-mediated heterogeneity of soil nitrogen and light at the forest floor. Ecological Research. 2003; 18(5):559–571.

[20] Griffiths RP, Madritcha MD, Swansona AK. The effects of topography on forest soil characteristics in the Oregon Cascade Mountains (USA): Implications for the effects of climate change on soil properties. Forest Ecology and Management. 2009; 257(1):1–7.

[21] Liu J, Yunhong T, Slik JWF. Topography related habitat associations of tree species traits, composition and diversity in a Chinese tropical forest. Forest Ecology and Management. 2014; 330:75–81.

[22] Shary PA, Smirnov NS. Mechanisms of the effects of solar radiation and terrain anisotropy on the vegetation of dark conifer forests in the Pechora–Ilych State Biosphere Reserve. Russian Journal of Ecology. 2013; 44(1):9–17.

[23] Velázquez-Rosas N, Meave J, Vázquez-Santana S. Elevation variation of leaf traits in montane rain forest tree species at La Chinantla, Sourthen Mexico. Biotropica. 2002; 34:534–546.

[24] Shary PA, Sharaya LS, Mitusov AV. Fundamental quantitative methods of land surface analysis. Geoderma. 2002; 107(1–2):1–32.

[25] Qi F, Zhu AX. Knowledge discovery from soil maps using inductive learning. International Journal of Geographical Information Science. 2003; 17:771–795.

[26] Schmidt J, Evans IS, Brinkmann J. Comparison of polynomial models for land surface curvature calculation. International Journal of Geographical Information Science. 2003; 17:797–814.

[27] Dikau R. The application of a digital relief model to landform analysis In Three dimensional applications in Geographical Information Systems, edited by Rapier J F, 51–77. London: Taylor & Francis, 1989.

[28] Bispo PC, Valeriano MM, Santos JR. Effects of the geomorphometric characteristics of the local terrain on floristic composition in the central Brazilian Amazon. Austral Ecology. 2012; 34(4):491–499.

[29] Jarvis A, Mulligan M. Terrain Controls on Tree Diversity and Structure in Tropical Forests: An Example in Two Neotropical Montane and Lowland Forests. London: VDM Verlag Dr. Müller; 2009.

[30] Bispo PC, Santos JR, Valeriano MM, Touzi R, Seifert FM. Integration of Polarimetric PALSAR Attributes and Local Geomorphometric Variables Derived from SRTM for Forest Biomass Modeling in Central Amazonia. Canadian Journal of Remote Sensing. 2014; 40:26–42.

[31] Webb EL, Stanfield BJ, Jensen ML. Effects of topography on rainforest tree community structure and diversity in American Samoa, and implications for frugivore and nectarivore populations. Journal of Biogeography. 1999; 26(4):887–897.

[32] Vitousek PM, Sanford RL. Nutrient cycling in moist tropical forest. Annual Review of Ecology and Systematics. 1986; 17:137–167.

[33] Kafkenscheid RLLJ. Hydrology and biogeochemistry of tropical montane rain forests of contrasting stature in the Blue Mountains. Jamaica. Thesis (PHD)—Vrije University, 302 pages; 2000.

[34] Aiba S, Kitayama K. Effects of the 1997–98 El Niño drought on rain forests of Mount Kinabalu, Borneo. Journal of Tropical Ecology. 2002; 18:215–230.

[35] Silver WL, Lugo AE, Keller M. Soil oxygen availability and biogeochemistry along rainfall and topographic gradients in upland wet tropical forest soils. Biogeochemistry. 1999; 44(3):301–328.

[36] Florinsky IV, Kuryakova GA. Influence of topography on some vegetation cover properties. Catena. 1996; 27(2):123–141.

[37] IBAMA (Instituto Brasileiro do Meio Ambiente e dos Recursos Naturais Renováveis) Floresta Nacional do Tapajós-Plano de Manejo. IBAMA, Belterra; 2004.

[38] Veloso R B, Rangel Filho ALR, Lima JCA. Classificação da vegetação brasileira, adaptada a um sistema universal Rio de Janeiro: IBGE,124 p.; 1991.

[39] RADAMBRASIL Departamento Nacional de Produção Mineral. Folha AS.21- Santarém. Geologia, geomorfologia, pedologia, vegetação e uso potencial da terra Rio de Janeiro: DNPM, 510 p. (Levantamento dos Recursos Naturais, v. 10); 1976.

[40] Gonçalves FG, Santos JR. Composição florística e estrutura de uma unidade de manejo florestal sustentável na Floresta Nacional do Tapajós, Pará. Acta Amazonica. 2008; 38(2):229–244.

[41] Galvão LS, Santos JR, Roberts DA, Breunig FM, Toomey M, Moura YM. On intra-annual EVI variability in the dry season of tropical forest: A case study with MODIS and hyperspectral data. Remote Sensing of Environment. 2011; 115(9):2350–2359.

[42] Frazer GW, Canham CD, Lertzman KP. Gap Light Analyzer (GLA), Version 2.0: Imaging software to extract canopy structure and gap light transmission indices from true-colour fisheye photographs, user’s manual and program documentation. Simon Fraser University, Burnaby, British Columbia, and the Institute of Ecosystem Studies, Millbrook, New York; 1999.

[43] Danson FM, Hetherington D, Morsdorf F, Koetz B, Allgöwer B. Forest Canopy Gap Fraction From Terrestrial Laser Scanning. IEEE Geoscience and Remote Sensing Letters. 2007; 4(1):157–160.

[44] Valeriano MM. Topodata: Guide for Use of Local Geomorphological Data. Documentation and Special Programs (INPE-15318-RPE/818). São José dos Campos: INPE: Coordination of Education; 2008.

[45] Valeriano MM, Rossetti DF. Topodata: Brazilian full coverage refinement of SRTM data. Applied Geography. 2012; 32:300–309.

[46] Valeriano MM, Albuquerque PCG. Topodata: processamento dos dados SRTM. São José dos Campos, SP: INPE: Coordenação de Ensino, Documentação e Programas Especiais (INPE-16702-RPQ/854). 79p.; 2010.

[47] Neter J, Kutner NH, Nac Hssheim CJ, Wasserman W. Applied Linear Statistical Models. 4 ed. Boston: McGraw Hill, 791pages; 1996

[48] Anselin L. The Moran scatterplot as ESDA tool to assess local instability in spatial association In: Fisher M, Scholten HJ, Unwin D (eds.). Spatial analytical perspectives on GIS. London: Taylor and Francis, p. 111–126; 1996.

[49] Bispo PC, Valeriano MM, Kuplich TM. Relation of local geomorphometric variables with the vegetation of the Madeira-Purus interfluve (AM/RO). Acta Amazonica. 2009; 39(1):81–90.

[50] Valeriano MM, Picini AG. Uso de Sistema de Informação Geográfica para a geração de mapas de médias mensais de temperatura do Estado de São Paulo. Revista Brasileira de Agrometeorologia. 2000; 8(2):255–262.

[51] Moore ID, Gessler PE, Nielsen GA, Peterson GA. Soil Attribute Prediction Using Terrain Analysis. Soil Science Society of America Journal. 1993;57(2):443–452.

[52] Seibert J, Stendahl J, Sørensen R. Topographical influences on soil properties in boreal forests. Geoderma. 2007; 141(1–2):139–148.

[53] Raghubanshi AS. Effect of topography on selected soil properties and nitrogen mineralization in a dry tropical forest. Soil Biology and Biochemistry. 1992; 24(2):145–150.

[54] Williams M, Shimabukuro YE, Herbert DA, Pardi La cruz S, Renno C, Rastetter EB. Heterogeneity of Soils and Vegetation in an Eastern Amazonian Rain Forest: Implications for Scaling Up Biomass and Production. Ecosystems. 2002; 5(7):692–704.

[55] Chauvel A., Lucas Y, Boulet R. 1987. On the genesis of the soil mantle of the region of Manaus, central Amazonia, Brazil. Experientia. 1987; 43:234–241.

[56] Castilho CV, Magnussona WE, Araújo RNO, Luizão RCC, Luizão FJ, Lima AP et al Variation in aboveground tree live biomass in a central Amazonian forest: Effects of soil and topography. Forest Ecology and Management. 2006; 234:85–96.

[57] Lambin EF, Geist H, Lepers E. Dynamics of land use and cover change in tropical regions. Annual Review of Environment and Resources. 2003; 28:205–241.

[58] Xin ZB, Xu JX, Zheng W. Spatio-temporal variations of vegetation cover on the Chinese Loess Plateau (1981–2006): Impacts of climate changes and human activities. Science in China Series D: Earth Sciences. 2008; 51 (1):67–78.

[59] Hunter MO, Keller M, Victoria D, Morton DC. Tree height and tropical forest biomass estimation. Biogeosciences. 2013; 10:8385–8399.

