# Correction: Predictive Models of Primary Tropical Forest Structure from Geomorphometric Variables Based on SRTM in the Tapajós Region, Brazilian Amazon

**DOI:** 10.1371/journal.pone.0155569

**Published:** 2016-06-08

**Authors:** The PLOS ONE Staff

The article’s citation appears incorrectly in the published article. The correct citation is: Bispo PC, Santos JR, Valeriano MM, Graça PMLA, Balzter H, França H, et al. (2016) Predictive Models of Primary Tropical Forest Structure from Geomorphometric Variables Based on SRTM in the Tapajós Region, Brazilian Amazon. PLoS ONE 11(4): e0152009. doi:10.1371/journal.pone.0152009.
