# Supplementary material for: Predictive Models of Primary Tropical Forest Structure from Geomorphometric Variables Based on SRTM in the Tapajós Region, Brazilian Amazon
Source: PLoS One. 2016 Apr 18;11(4):e0152009. doi: 10.1371/journal.pone.0152009 (PMC4835096; doi:10.1371/journal.pone.0152009)
Supplement: S1 File — (DOCX) [file pone.0152009.s001.docx]

| 1. BA | 1. CO |
| --- | --- |
|  |  |
|  | |

1. H

Spatial correlogram of the residual of the models built for BA (basal area), CO (canopy openness) and H (height). The Moran Index was not significant (p> 0.05) for any distance class.
